# Beyond Enumeration: Functional and Computational Analysis of Circulating Tumor Cells to Investigate Cancer Metastasis

**DOI:** 10.3389/fmed.2018.00034

**Published:** 2018-02-19

**Authors:** Francesc Castro-Giner, Manuel C. Scheidmann, Nicola Aceto

**Affiliations:** ^1^Department of Biomedicine, Cancer Metastasis Laboratory, University of Basel, University Hospital Basel, Basel, Switzerland; ^2^Swiss Institute of Bioinformatics, Lausanne, Switzerland

**Keywords:** circulating tumor cells, molecular analysis, computational biology, metastasis, single-cell genomics

## Abstract

Circulating tumor cells (CTCs) are defined as those cells that detach from a cancerous lesion and enter the bloodstream. While generally most CTCs are subjected to high shear stress, anoikis signals, and immune attack in the circulatory system, few are able to survive and reach a distant organ in a viable state, possibly leading to metastasis formation. A large number of studies, both prospective and retrospective, have highlighted the association between CTC abundance and bad prognosis in patients with various cancer types. Yet, beyond CTC enumeration, much less is known about the distinction between metastatic and nonmetastatic CTCs, namely those features that enable only some CTCs to survive and seed a cancerous lesion at a distant site. In addition, critical aspects such as CTC heterogeneity, mechanisms that trigger CTC intravasation and extravasation, as well as vulnerabilities of metastatic CTCs subpopulations are poorly understood. In this short review, we highlight recent studies that successfully adopted functional and computational analysis to gain insights into CTC biology. We also discuss approaches to overcome challenges that are associated with CTC isolation, molecular and computational analysis, and speculate regarding few open questions that currently frame the CTC research field.

## Introduction

More than 90% of cancer-related deaths are due to metastasis development (1). However, historically, the majority of cancer research has been conducted with a focus on the primary tumor, mostly because of the higher availability of primary tumor specimens compared to biopsies of metastatic lesions, and difficulties to investigate spontaneous metastatic dissemination *in vivo* (2). As a consequence, our understanding of the vulnerabilities of metastatic cells remains limited, thus hampering the development of effective metastasis-suppressing agents. The metastatic process is commonly thought to be a relatively inefficient, multistep phenomenon that involves the intravasation of cancer cells into the blood circulatory system, their survival in the blood as circulating tumor cells (CTCs), followed by extravasation and seeding of metastatic CTCs into distant sites (3). While it is generally accepted that tumors are able to shed a relatively high number of cancer cells in circulation, it also appears that most CTCs are poised to die in the bloodstream or upon arrival to a distant site, mostly due to high shear forces and anoikis signals in circulation, immune attack or limited capability to adapt to a foreign microenvironment (4). It is therefore critical to dissect features that distinguish those CTCs that are able to survive and initiate metastasis. To this end, liquid biopsy (e.g., blood sampling) represents a minimally invasive yet extraordinary valuable source of CTCs (as well as other tumor-derived material such as circulating tumor DNA, proteins, and exosomes) from virtually all cancer types (5).

### CTC Isolation from Blood Samples

In recent years, we have witnessed remarkable improvements in the ability to efficiently isolate CTCs from blood samples. Several CTC isolation technologies are now available and designed to overcome constrains such as CTCs dilution in blood samples and variations in expression levels of cell surface markers that distinguish CTCs from blood cells. While these technologies have been extensively discussed elsewhere (6–10), on the other hand, it is becoming increasingly clear that a consensus is not yet reached within the CTC community regarding which technology should be used as a reference for CTC-related studies. While the CellSearch system has been FDA-cleared for CTC enumeration, a gold-standard technology for live CTC isolation and molecular analysis is not yet defined. Presently, it is often the case that CTC isolation for molecular catheterization is conducted in different laboratories with different technologies, and this may result in biases such as the preferential entrapment of specific CTC subpopulations depending on the technology of choice. Future studies involving large sample numbers will be needed to address specific biases of current individual technologies and indicate a reference tool for CTC isolation to standardize CTC-related studies.

While challenging and still subjected to technology-driven biases, CTC isolation and molecular characterization offers an extraordinary opportunity to investigate the metastatic process in real time and with minimally invasive procedures (i.e., blood sampling). Molecular analysis of CTCs often implies dealing with very limited cell numbers (10) and adapting molecular assays and computational analysis tools toward single-cell resolution (11). In the next paragraphs, we discuss recent studies that adopted molecular and computational analysis of CTCs to dissect features of metastatic cells. We also discuss single-cell analysis-related challenges that are typically encountered in CTC studies.

## Molecular Analysis of CTCs

Molecular interrogation of CTCs has been made possible not only by the development of specialized CTC isolation technologies but also with the achievement of single-cell-resolution-sequencing protocols (12) and single-cell-based assays (13, 14). The application of these approaches to CTCs has already generated a number of exciting observations, many of which provide insightful information in regard to features of metastatic precursors, CTC heterogeneity, and patient stratification.

### Metastatic Precursors among CTCs

Circulating tumor cells are found in the blood of patients as single cells and as clusters of cells, with the latter being associated to a higher metastatic potential (15–19). Using mouse models with multicolor primary tumors, CTC clusters have been shown to break off from tumors already as clusters and to be composed by an oligoclonal group of cells (18). The oligoclonality of CTC clusters might have relevant implications regarding the fitness, seeding capability, and potential to resist to therapy of cluster-derived metastatic foci; yet, this has to be further investigated. RNA sequencing of CTC clusters from breast cancer patients showed that cells within clusters rely upon the expression of cell–cell junction components such as plakoglobin, and that targeting plakoglobin reduces CTC clustering and metastasis in mouse models (18). Beyond plakoglobin—which is a challenging pharmacological target—it is currently unclear how to prevent or suppress the formation of CTC clusters *in vivo*, and further studies will be needed to identify cluster-specific vulnerabilities, as well as—more broadly—the vulnerabilities of metastatic precursors.

### CTC Heterogeneity

Various studies have tackled the issue of CTC heterogeneity. For example, the phenotypic analysis of CTCs from patients initially diagnosed with ER-positive/HER2-negative breast cancer showed that CTCs are able to acquire HER2 expression in the metastatic setting (20). Using a combination of CTC culturing, single-cell RNA sequencing (scRNA-seq), and a small-scale drug screen combined with mass spectrometry analysis, it was shown that HER2-positive CTCs are more proliferative but not addicted to HER2, while HER2-negative CTCs display the activation of Notch and DNA damage pathways, as well as resistance to cytotoxic therapy (20). Combination treatment with paclitaxel and Notch inhibitors enables the suppression of the tumorigenic potential of both HER2-negative and HER2-positive phenotypes (20). In a separate study, the phenotypic analysis of human breast CTCs using quantitative RNA *in situ* hybridization (RNA-ISH) revealed a dynamic expression of epithelial versus mesenchymal markers, mostly as a consequence to treatment (21), suggesting that treatment itself may strongly impact the phenotypic heterogeneity of CTCs. In prostate cancer, the RNA expression profile of CTCs revealed a high degree of intra-patient heterogeneity, but even higher diversity across CTCs from different patients (22). For instance, prostate CTCs were shown to contain diverse AR gene mutations and splicing variants, with the activation of Wnt-signaling pathway featuring a subgroup of CTCs belonging to patients who were resistant to anti-androgen therapy (22). Among others, examples of CTC heterogeneity include studies showing Wnt2 expression mediating metastasis-associated survival signals through the TGF-b-activated kinase 1 in a subset of pancreatic CTCs (23), as well as evidence that the expression of specific gene combinations—such as HER2/EGFR/HPSE/Notch1 and EpCAM/CD44/CD47/MET—may confer high metastatic potential to breast CTCs (24, 25).

### Patient Stratification through the Analysis of CTCs

Molecular analysis of CTCs has also recently provided very exciting observations regarding patient stratification and the use of CTCs as a surrogate tool to investigate tumor genotypes and response to therapy. In multiple myeloma, when analyzing the mutational landscape of CTCs and matched bone marrow tumor cells, it was found that alterations in oncogenes like KRAS, NRAS, or BRAF were detectable in both specimens, suggesting blood sampling as a reliable and less invasive method for mutational screening of such patients (26). In a similar fashion, comparing whole-exome sequencing data with primary tumor, matched lymph node metastasis and CTCs from a prostate cancer patient revealed that the majority of CTC mutations were present in matched tumor tissue, thus providing a proof-of-concept for the effectiveness of CTC genomics in the clinical setting (27). With regard to patient stratification, CTCs have also proven useful to stratify chemosensitive versus chemorefractory small-cell lung cancer patients (28). Particularly, it was shown how DNA copy number variations (CNVs) can be assessed in small-cell lung CTCs and help predict responsiveness to chemotherapeutic agents, arguing that CTC analysis may be used as a tool to guide treatment decisions in the clinical setting (28). In addition to the analysis of CTCs, interrogation of other blood components including circulating tumor DNA (currently more advanced on the clinical side) allows to detect cancer-associated variants in the blood of patients with good sensitivity and specificity; yet, it may require a more targeted approach (i.e., assessing specific hotspots that are known or expected to carry a mutation) compared to CTC analysis.

Taken together (see also Table 1), these examples of molecular CTC analysis represent a proof of concept of the versatility and potential of CTC-related studies to investigate the metastatic process as well as to influence treatment decisions, complementarily to the analysis of other tumor-derived blood components such as circulating tumor DNA, reviewed elsewhere (5). Hand in hand with the molecular characterization of CTCs, computational analysis represents a major accelerator to take full advantage of CTC-sequencing efforts and to generate actionable hypotheses. Below, we discuss typical challenges of computational CTC data analysis, mostly dealing with single-cell resolution data, and approaches to help overcome current sequencing limitations.

**Table 1:** Examples of adopted methods to enable a molecular analysis of circulating tumor cells (CTCs).

Molecular assay	Target	Reference
Tracing fluorescently labeled cancer cells in circulation	Quantification of the metastatic potential of single CTCs and CTC clusters	(18, 19)
Quantitative mass spectrometry of cultured CTCs	Detection of protein expression levels to identify differentially regulated proteins upon drug treatment	(20)
Quantitative RNA *in situ* hybridization	Assessment of dynamically expressed transcripts upon drug treatment and identification of CTC subpopulations	(21, 25)
Direct xenograft transplantation of patient-derived CTCs	Phenotypic analysis of metastasis-initiating CTCs	(24)
Single-cell RNA sequencing	Detection of gene expression changes to identify differentially regulated genes and pathways in individual CTCs	(18, 20, 22)
Single-cell DNA sequencing	Identification of single nucleotide variants (SNPs), insertions, deletions, amplifications, and translocations to determine the genomic landscape of individual CTCs	(26–28)

*The molecular assay types, their main objective (target), and the corresponding references are summarized*.

## Computational Analysis of CTC

With a number of recent technological breakthroughs in cell capture and single-cell-sequencing (SCS) protocols, it is now possible to interrogate the genome, transcriptome, and epigenome of CTCs. The application of these technologies is improving our understanding of CTC biology as well as the relationship between CTCs and matched primary or metastatic tumors (12). Yet, despite significant improvements in SCS, the major analytical challenges remain. The main challenge is the interpretation of data in the context of strong stochastic variation and high error rate generated during the amplification of the low amount of starting material derived from a single cell. To address this issue, noise-specific computational methods have been developed. In the following paragraphs, we summarize the current challenges in the analysis of SCS data, with an emphasis on those that are potentially affecting the interpretation of CTC studies (see also Table 2).

**Table 2:** Computational methods for circulating tumor cell (CTC) analysis based on single-cell-sequencing approaches.

Computational method	Technical challenges	Target	Reference
Single-cell DNA sequencing	– Low coverage – Nonuniform coverage – PCR errors – Allele dropout – Allelic imbalance	SNV	(39)
		CNV	(42–46)
		Phylogeny	(51, 52, 54–56)

Single-cell RNA sequencing	– Amplification bias – Dropout of low abundant transcripts	Gene expression profiling	(60–62, 67, 69)
		Differential expression	(63, 64, 68)
		Coexpression network	(76)
		Phylogeny	(47, 48, 69–75)
		SNV	(49)
		CNV	(47, 48)

*Computational methods for CTC analysis, related technical challenges and applications (target), as well as references are shown*.

*SNV, single nucleotide variant; CNV, copy number variant*.

As a matter of fact, there are specific aspects of CTCs that might contribute to increase the noise of the data. First, CTCs are exposed to different extrinsic stress factors *in vivo*, such as shear stress, attack by the immune system, and anticancer therapies (10, 29). These phenomena might modify the gene expression profile and induce apoptosis, thus reducing the quality and quantity of the extracted DNA and mRNA. Second, depending on the method used for isolation, CTCs can be admixed with leukocytes, stromal cells, and platelets (30). In RNA-seq data, it is possible to perform a negative selection of samples that show a substantial contamination, for instance, by evaluating the expression of specific leukocyte-associated markers or to digitally remove signatures of genes typically belonging to benign cells (31). In addition, CTC can be found as a single cell or clusters of cells (18). When not carefully controlled for, this introduces variability on the amount of starting material per each sample, possibly leading to systematic biases during amplification steps.

As described below, noise and low coverage on SCS analysis is traditionally compensated by combining data from other cells, usually from dozens to thousands of cells. However, the dilution factor of CTCs in the bloodstream and the limited volume of blood available from cancer patients generally lead to obtain only very few cells per patient (typically between 0 and 10 per tube of blood) (11). This limitation is traditionally addressed by pooling cells from the same individual, but this hampers any conclusions regarding the CTC heterogeneity and the detection of rare CTC subpopulations. Experimental solutions such as CTC-derived explants and the development of cell lines from captured CTC can help to increase the number of cells to investigate (32, 33), at the expense of low efficiency of such processes and possible biases during *ex vivo* culturing.

### Variant Detection

Single-cell DNA sequencing (scDNA-seq) of CTCs followed by mutation calling has enabled to investigate oligoclonality in CTCs and to identify differences compared to primary and metastatic tumors (27, 34–36). Yet, the data obtained after whole-genome amplification (WGA) is generally characterized by low-coverage breath, nonuniform coverage, false-positive (FP) errors introduced by PCR, false-negative (FN) errors due to insufficient coverage, allele dropout (ADO), and allelic imbalance. To date, there are no computational approaches for variant detection that model this noise. As an alternative, most published studies tried to estimate technical variability comparing the variants obtained from single-cell data with bulk sequencing or control samples (27, 37, 38). Currently, only single nucleotide variants (SNVs) and CNVs can be detected accurately from SCS.

Methods developed for SNV detection (39) rely on the mutation frequency across cells to calculate the posterior probability of the variant to be present in at least two cells. This approach reduces the fraction of FP but, as a consequence, mutations observed in only one cell are discarded. More robust methods will be needed to address FN events introduced by ADO, the major contributor to technical errors, affecting 10–50% of mutation sites (40, 41). Current methods for CNV use segmentation algorithm based on GC-normalized coverage (42–46). These methods are heavily affected by the nonuniform coverage obtained after WGA. Although this can be partially improved using amplification protocols that produce more uniform coverage (40), the resolution is limited to megabase-scale CNVs (42, 44). On the other hand, these methods have shown a relatively high specificity in low-coverage data (42, 44, 46). Further, variant detection can also be performed on scRNA-seq data, and a number of studies have attempted to infer copy number profiles by averaging the relative expression levels over large genomic regions (47, 48). RNA-seq can be used for SNV calling, but this approach is limited by RNA-editing events, allele-specific expression, and the small fraction of genes that are expressed at a high level (49).

A specific downstream analysis of variant calling is the reconstruction of single-cell phylogenies. These phylogenies can reconstruct the subclonal compositions of cells, revealing the evolutionary history of the tumor and, in the case of CTC-sequencing studies, specific metastatic-seeding patterns. A variety of approaches have been recently developed that are customized for the characteristics of scDNA-seq data (50–55). Some of these models can accommodate doublet cells into the analysis (51, 56) and are of particular interest for the analysis of CTC clusters. At the moment, current approaches only use SNVs as source information, and additional efforts are required to integrate CNV into the models.

### Gene Expression Profiling

Methods of whole transcriptome amplification (WTA) for single cells are well established but also suffer from amplification bias and unwanted variation. To overcome this challenge, some of these protocols integrate unique molecular identifiers that allow to track single molecules through the amplification process, thus reducing amplification bias (57). In addition, spike-ins of known concentration can be used to quantify sensitivity and technical variation (58). However, WTA and subsequent analysis methods struggle with reliable amplification for lowly expressed transcripts (59), leading to dropout events that add uncertainty to downstream analyses. Different methods and workflows have been developed for single-cell quality control that allows to remove batch effects and unwanted biological variability such as cell-cycle variation (60–62). To compare expression across cells, the data need to be normalized to remove cell-specific biases such as read depth and cell capture efficiency. Specific methods are available to normalize scRNA-seq data that account for a high degree of technical noise, low coverage, and a high proportion of dropout events (63–65).

Generally, there are two main downstream analyses of scRNA-seq data: differential expression (DE) and clustering. DE may help to find specific expression signatures associated with metastasis (31, 66) or to study differences between CTC and CTC clusters (18). It is possible to use methods for DE developed for bulk sequencing (67) coupled with the single-cell-specific pre-normalization. However, there are tools specifically developed for scRNA-seq, such as SCDE (64), MAST (63), and BASiC (68), that apply their specific normalization approaches. Second, similar to scDNA-seq phylogenies, unsupervised clustering of cells based on scRNA-seq can help to identify differences across cells and subclonal tumor populations. There is a wide range of methods available for clustering developed for scRNA-seq data, each of them with specific advantages and disadvantages (47, 48, 69–75). Besides DE and clustering, scRNA-seq has been used for network modeling to provide insights about complex transcriptional regulation. The most popular approach that has been already applied in a number of scRNA-seq studies is gene coexpression network analysis (WGCNA) (76). However, network analysis methods require a large sample size, which rarely applies to CTC studies.

### Single-Cell Multi-omics

Recent technological breakthroughs now allow simultaneously interrogating RNA and DNA from a single cell, enabling a parallel analysis of gene expression, methylation status, and DNA mutations (77–81). This opens up the possibility to more comprehensively understand cellular processes. For example, it is now feasible to correlate DNA methylation status with gene expression and to directly link genomic variation to transcriptional variability to discover expression quantitative loci (79, 82). To date, there are no computational methods that are tailored to combine multiple molecular layers and at the same time to control for technical variation from single-cell genomics. Computational tools in this field are needed, as future developments in amplification and sequencing techniques will allow to robustly apply single-cell multi-omics to study complex questions in CTC biology.

## Concluding Remarks

We have recently witnessed extraordinary advances in understanding CTC biology, including the potential of CTCs to reveal targetable cancer vulnerabilities. For instance, several proof-of-concept studies have begun dissecting the heterogeneity of CTCs and have highlighted characteristics that enable few selected CTCs to seed a metastasis. Other studies have shown a potential for CTCs to drive patient stratification and treatment eligibility choices. However, many questions remain to be answered, several of which relate to the applicability of CTC analysis in the clinical setting (possibly together with the analysis of circulating tumor DNA) and the identification of metastasis-suppressing therapies. To this end, computational analysis plays a pivotal role. Addressing major single-cell-related challenges in computational biology will allow scientists to generate hypotheses and interpret data in a highly reliable manner and to accelerate the discovery process in CTC biology and beyond.

## Author Contributions

All authors have contributed to write the manuscript and approve its final version.

## Conflict of Interest Statement

NA is listed as an inventor in patent WO2015061091 on “Treating cancer, by measuring level of CTC clusters in sample obtained from patient with breast or epithelial cancer, administering treatment to prevent or reduce metastasis” owned by the Massachusetts General Hospital, Boston, MA, USA, and patent PCT/EP2017/068024 on “Apparatus and methods for disrupting circulating tumor cell clusters” owned by the University of Basel, the University of Linz, and Griesmühle Kleinkraftwerk GmbH. All the other authors declare no potential conflict of interest.
